# Post-marketing safety profile of lumacaftor/ivacaftor in cystic fibrosis treatment: a pharmacovigilance analysis based on FAERS

**DOI:** 10.3389/fmed.2026.1750064

**Published:** 2026-02-23

**Authors:** Tao Wang, Weilun Yang, Ye Luo, Yue Zhan, Fan Zou, Zhiwei Cui, Yuanbo Lan

**Affiliations:** 1 Department of Respiratory and Critical Care Medicine, Affiliated Hospital of Zunyi Medical University, Zunyi, China; 2 Department of Tuberculosis, Affiliated Hospital of Zunyi Medical University, Zunyi, Guizhou, China; 3 Department of Obstetrics and Gynecology, The First Affiliated Hospital of Xi’an Jiaotong University, Xi’an, China; 4 Guizhou Provincial Key Laboratory of Pathogenesis and Prevention of Common Chronic Diseases, Zunyi, China

**Keywords:** adverse drug events, cystic fibrosis, depression, disproportionality analysis, lumacaftor/ivacaftor

## Abstract

**Background:**

Lumacaftor/ivacaftor (LUM/IVA) is a CFTR modulator approved for the treatment of cystic fibrosis (CF) caused by the F508del mutation. While clinical trials have demonstrated its efficacy, the long-term safety profile remains inadequately characterized. This study aims to assess the real-world safety of LUM/IVA through a comprehensive analysis of adverse drug events (ADEs) reported in the U. S. FDA Adverse Event Reporting System (FAERS) from the third quarter of 2015 to 2024.

**Methods:**

Disproportionality analysis including the reporting odds ratio (ROR) and Bayesian confidence Propagation Neural Network (BCPNN) was employed to identify potential safety signals associated with LUM/IVA. Furthermore, sensitivity analyses were conducted to eliminate potential confounding arising from concomitant medication use and underlying indications. Potential risk factors for LUM/IVA-associated depression were identified through logistic regression analysis. Finally, time-to-onset (TTO) analysis was conducted to evaluate the temporal patterns of ADEs.

**Results:**

A total of 7,843 LUM/IVA-related ADE reports were extracted, spanning 27 system organ classes (SOCs). We identified 221 positive signals, with 37 signals consistent with the drug label, 61 signals might be associated with disease progression, and 123 signals not listed in the drug label. Positive signals with most reported cases included infective pulmonary exacerbations (*n* = 1,394, ROR 1201.58, IC025 9.50), hospitalization (*n* = 841, ROR 20.07, IC025 4.13) and, dyspnoea (*n* = 517, ROR 4.00, IC025 1.83). Unexpected signals such as depression (*n* = 81, ROR 1.71, IC025 0.45), suicidal ideation (*n* = 31, ROR 1.63, IC025 0.20), and hypoglycemia (*n* = 20, ROR 2.02, IC025 0.39) were detected, highlighting previously unreported risks. Stratified analysis identified risk signals in different subgroups, such as chest discomfort in 50–100 kg subgroup. The TTO analysis revealed that the median onset of ADEs was 256 days, with most ADEs reports occurring after 360 days of treatment LUM/IVA (39.2%), Moreover, cumulative incidence varied significantly among subgroups stratified by age, dosage, and frequency of administration.

**Conclusion:**

This study provides a detailed real-world safety profile of LUM/IVA, identifying both known and unexpected adverse events. Our findings underscore the importance of continuous post-marketing surveillance and the need for updated drug label to reflect associated risks, particularly those related to psychiatric symptoms and long-term adverse effects. Personalized treatment strategies based on patient characteristics, including sex, are recommended to optimize safety during LUM/IVA therapy.

## 1 Background

Cystic fibrosis (CF) is an autosomal recessive genetic disorder primarily caused by mutations in the CF transmembrane conductance regulator (CFTR) gene. The most common CFTR mutation is the Phe508del mutation (1). The loss of CFTR function leads to impaired chloride and bicarbonate transport in epithelial cells, resulting in chronic airway infections and neutrophilic inflammation (2), ultimately causing damage to the airway walls and bronchiectasis. Approximately 162,428 individuals worldwide are affected by CF, with around 65% of them diagnosed (3). CFTR modulators have been approved in several countries for the treatment of CF. This therapy is considered a lifelong transformative treatment (4), closely associated with significant improvements in patients’ quality of life and extended life expectancy. The lumacaftor/ivacaftor (LUM/IVA) combination, marketed under the brand name Orkambi^®^, has been approved for use in patients with F508del homozygous cystic fibrosis, for children aged 1 year and older (5). The potentiator ivacaftor works by binding directly to the CFTR channel, increasing its probability of opening, while the corrector lumacaftor acts as a pharmacological chaperone, promoting the proper folding of the CFTR protein, thereby enhancing its transport to the apical cell membrane (6). Clinical trial results have shown that the rate of pulmonary function deterioration in the LUM/IVA group was 30–39% lower than in the placebo group, with a significant reduction in hospitalizations and the need for intravenous antibiotics (7). Results from three additional phase III clinical trials, targeting different age groups, further support the long-term efficacy of LUM/IVA (8–10).

Although LUM/IVA has been proven effective in clinical practice, reports of adverse drug events (ADEs) have gradually increased due to the possibility of long-term use. A case–control study involving 105 CF patients indicated that among the 72 patients treated with LUM/IVA, adverse reactions were more commonly reported, with 55% of patients experiencing chest tightness or shortness of breath, and 32% discontinuing treatment as a result (11). Another study also showed that patients with severe lung disease treated with LUM/IVA commonly experienced respiratory-related adverse events, including chest tightness and shortness of breath (12). A phase II open-label clinical trial targeting children revealed that most treatment-related adverse events were assessed as mild (38.8%) or moderate (40.8%) (13). However, the inherent limitations of clinical trials, such as strict inclusion criteria, relatively small sample sizes, and limited follow-up time, restrict their ability to fully capture real-world safety data. Therefore, post-marketing pharmacovigilance studies are essential for the broad monitoring and analysis of ADEs, ensuring the long-term safety of the drug.

The U. S. FDA Adverse Event Reporting System (FAERS) is a globally leading spontaneous reporting database that plays a critical role in post-marketing safety surveillance of approved drugs. This system consolidates adverse event reports voluntarily submitted by healthcare professionals, pharmacists, drug manufacturers, and consumers from the United States and other countries. By utilizing advanced data mining algorithms, it enables the quantitative assessment of potential associations between drugs and ADEs, providing valuable support for pharmacoepidemiological studies and pharmacovigilance analyses (14). This study aims to evaluate the real-world safety of LUM/IVA by analyzing FAERS data. Our findings will provide comprehensive insights into the safe use of LUM/IVA and offer guidance for clinical personalized drug selection.

## 2 Methods

### 2.1 Data cleaning and deduplication process

**Figure 1:**
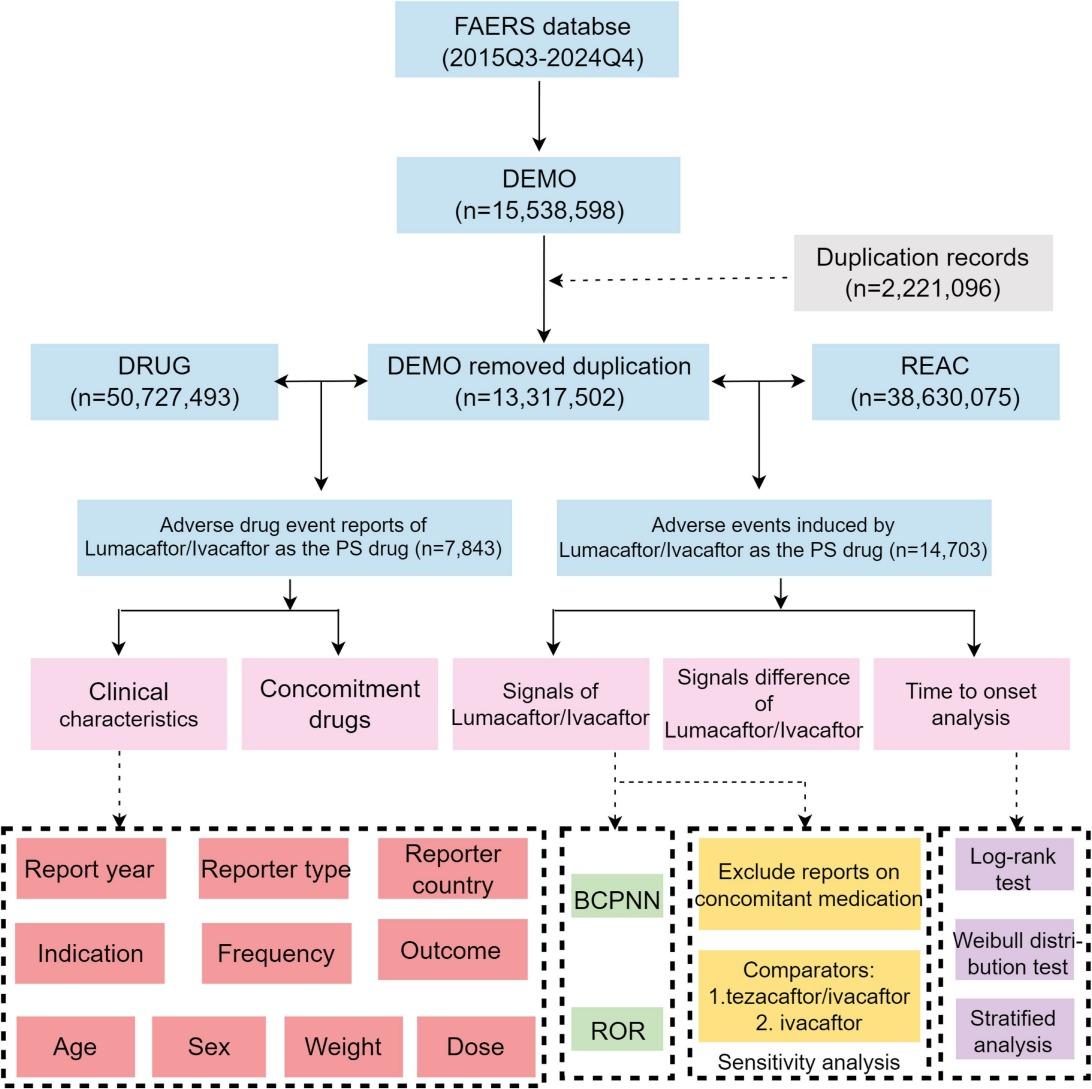
Flowchart of the whole research. FAERS, The U. S. FDA Adverse Event Reporting System; PS, primary suspect; ROR, reporting odds ratio; BCPNN, Bayesian confidence propagation neural network; Q3, third quarter; Q4, fourth quarter.

FAERS represents the largest pharmacovigilance database globally, compiling ADEs from diverse sources, including healthcare professionals, pharmaceutical manufacturers, and patients (15). FAERS enables systematic monitoring and evaluation of drug safety, facilitating the identification of potential risks and benefits associated with medication use. It is an essential resource for pharmacovigilance research. For this study, we extracted data from the FAERS database covering the period from the third quarter (Q3) of 2015—following FDA approval of LUM/IVA—to the fourth quarter (Q4) of 2024. FAERS comprises seven standardized tables: DEMO (demographics), DRUG (drug information), INDI (indications), REAC (reactions), OUT (outcomes), RPSR (report sources), and THER (therapy start and end dates). Deduplication was performed in accordance with FDA-recommended procedures (16). Specifically, DEMO records were sorted by CASEID and FDA_DT, retaining only the most recent FDA_DT entry per CASEID. In instances of identical FDA_DT values, the record with the highest PRIMARYID was retained. For data from Q4 2019 onward, records listed in the “deleted case” files were excluded (17). To identify relevant drug exposures, we queried both the generic name (lumacaftor/ivacaftor; VX-809 VX-770 combination) and the brand name (ORKAMBI). Only records in which the drug was designated as the primary suspect (PS) in the DRUG table were included in the analysis (18). ADEs were coded using the Medical Dictionary for Regulatory Activities (MedDRA), version 27.1 (19). Both preferred terms (PTs) and system organ classes (SOCs) were utilized to standardize and categorize AE terms for analysis. Our analytical workflow is shown in Figure 1.

### 2.2 Study population

This study was a retrospective pharmacovigilance analysis based on individual case safety reports retrieved from FAERS. The study population consisted of reports submitted between Q3 of 2015 and Q4 of 2024. Reports were eligible for inclusion if LUM/IVA (including generic names and the brand name) was identified as the PS drug. No restrictions were imposed on age, sex, or geographic origin, in order to capture a comprehensive real-world safety profile. Reports involving duplicate case identifiers were removed according to FDA-recommended deduplication procedures. Given the nature of spontaneous reporting systems, incomplete information on demographic variables (such as age, body weight, and dosing details) was permitted, and analyses were conducted based on available data. Subgroup analyses, including those stratified by age, weight, dosing frequency, and sex, were restricted to reports with complete information for the relevant variables.

### 2.3 Disproportionality analysis

To identify potential safety signals associated with LUM/IVA, we conducted disproportionality analysis, a well-established and widely accepted method in pharmacovigilance for signal detection (20). This approach assesses whether specific ADEs are reported more frequently than expected by comparing the observed and expected reporting frequencies, thereby quantifying the strength of association between a drug and an ADE. Two disproportionality methods were employed: the reporting odds ratio (ROR), a frequentist approach that provides interpretable estimates of relative risk, and the Bayesian confidence Propagation Neural Network (BCPNN), which combines Bayesian inference with neural network-based learning to enhance sensitivity and reduce bias, particularly in the presence of sparse data (21). Given the distinct methodological strengths of each approach and the absence of consensus on a universally superior method, both were applied in parallel to ensure analytical robustness. To minimize the risk of false positives and strengthen the validity of detected signals, only ADEs that simultaneously met predefined statistical significance thresholds for both ROR and BCPNN were considered positive signals and retained for further analysis (22). All analyses were performed based on 2 × 2 lists (Table 1).

**Table 1:** Disproportionality analysis methods and thresholds for ROR and BCPNN.

Drug category	Target adverse drug event	Non-target adverse drug event	Sums
lumacaftor/ivacaftor	a	b	a + b
Non-lumacaftor/ivacaftor	c	d	c + d
Total	a + c	b + d	a + b + c + d

Methods	Formula	Threshold
ROR	$\mathrm{ROR}=\frac{a/c}{b/d}$	a≥3
	$\mathrm{SE}(\text{lnROR})=\sqrt{\left(\frac{1}{a}+\frac{1}{b}+\frac{1}{c}+\frac{1}{d}\right)}$	
	$95\%\mathrm{CI}=e^{\ln(\mathrm{ROR})\pm 1.96\mathrm{se}}$	95%CI(lower limit)>1
BCPNN	$\mathrm{IC}=\log_{2}\frac{p(x,y)}{p(x)p(y)}=\log_{2}\frac{a(a+b+c+d)}{\left(a+b)(a+c\right)}$	IC025>0
	$E(\mathrm{IC})=\log_{2}\frac{\left(a+\gamma 11)(a+b+c+d+\alpha )(a+b+c+d+\beta \right)}{\left(a+b+c+d+\gamma )(a+b+\alpha 1)(a+c+\beta 1\right)}$	
	$V(\mathrm{IC})=\frac{1}{{\left(\ln2\right)}^{2}}[\frac{(a+b+c+d)-a+\gamma -\gamma 11}{\left(a+\gamma 11)(1+a+b+c+d+\gamma \right)}+\frac{(a+b+c+d)-(a+b)+a-\alpha 1}{\left(a+b+\alpha 1)(1+a+b+c+d+\alpha \right)}+\frac{(a+b+c+d+\alpha )-(a+c)+\beta -\beta 1}{\left(a+b+\beta 1)(1+a+b+c+d+\beta \right)}]$	
	$\gamma =\gamma 11\frac{\left(a+b+c+d+\alpha )(a+b+c+d+\beta \right)}{\left(a+b+\alpha 1)(a+c+\beta 1\right)}$	
	$\mathrm{IC}-2\mathrm{SD}=E(\mathrm{IC})-2\sqrt{V(\mathrm{IC})}$	

a, the number of reports involving both lumacaftor/ivacaftor and the target adverse event; b, the number of reports involving lumacaftor/ivacaftor and other adverse events; c, the number of reports involving the target adverse event with other drugs; and d, reports involving other drugs and non-target adverse events. ROR, reporting odds ratio; BCPNN, Bayesian confidence Propagation Neural Network; 95% CI, 95% Confidence Interval; χ^2^, Chi-squared statistic; IC, Information Component; IC025, 2.5th percentile of the IC; E(IC), Expected IC; V(IC), Variance of IC.

### 2.4 Clinical priority scoring

To enhance the clinical relevance of detected safety signals, all positive AEs were subjected to a structured clinical prioritization assessment encompassing four key dimensions (Table 2): (i) reporting frequency, (ii) signal stability across time, (iii) mortality rate among reported cases, and (iv) clinical relevance based on medical judgment and pathophysiological plausibility (23, 24). Each dimension was independently scored, and the total score was used to categorize signals into three priority levels: low (0–2 points), medium (3–5 points), and high (6–8 points). This multidimensional evaluation framework enabled a more nuanced interpretation of pharmacovigilance data, ensuring that downstream analyses and risk communication efforts remained focused on signals of greatest clinical and regulatory importance.

**Table 2:** Criteria and scoring framework for prioritizing ADEs identified via disproportionality analysis.

Criterium	2 points	1 point	0 point
Reporting rate (cases/non-cases)	>10%	1–10%	0–1%
Magnitude of the lower limit of the 95% CI of the ROR	>5	2–5	1–2
Reported case fatality rate (proportion of reports with death as outcome)	>50%	25–50%	<25%
Clinical relevance (serious likely drug-attributable ADEs)	DME	IME	None

ROR, reporting odds ratio; DME, designated medical event; IME, important medical event. ADEs, adverse drug events, DME designated medical event, IME important medical event.

### 2.5 Sensitivity analyses

Two sensitivity analyses were conducted to assess the robustness of the results: (1) Concomitant medication exclusion—Reports involving the concomitant use of LUM/IVA and other medications were excluded, and the disproportionality analysis was re-executed to evaluate the signal strength (25). (2) Indication-specific comparators analysis—To address the possibility that certain ADEs may be related to CF progression rather than drug exposure, the analysis was restricted to patients receiving CFTR modulators. The ROR and Information Component (IC) values for tezacaftor/ivacaftor/ivacaftor (Symdeko) and ivacaftor (Kalydeco), both indicated for CF, were recalculated and used as comparators to Orkambi (22).

### 2.6 Modified ROR algorithm for stratified analysis

To investigate subgroup-risk signals, we applied a modified ROR algorithm informed by previous pharmacovigilance research (26). As an example, we present the sex-stratified subgroup analysis. In this subgroup analysis, a 2 × 2 contingency table was constructed as follows: a = number of target ADE reports in males; b = number of other ADE reports in males; c = number of target ADE reports in females; d = number of other ADE reports in females. Although this approach does not represent a conventional epidemiological ROR, it provides a comparative measure of disproportionality across sex subgroups. Statistical inference was based on 95% confidence intervals and adjusted *p*-values (*P*.adj). A lower confidence interval bound >1 with *P*.adj < 0.05 indicated a risk signal in males, whereas an upper bound <1 with *P*.adj < 0.05 indicated a risk signal in females.

### 2.7 Logistic regression analysis

Given that depression emerged as a potential safety signal in the disproportionality analysis, we conducted both univariate and multivariate logistic regression to identify independent risk factors associated with its reporting. A total of 3,440 complete reports containing information on age, sex, reporter type, reporting country, treatment frequency, and concomitant medication status were included in the analysis. Variables with a *p* < 0.05 in univariate analyses were entered into the multivariate model, which was fitted using the “rms” package (version 6.4.0) in R.

### 2.8 Time to onset and stratified analysis

Time to onset (TTO) for each ADE reports was calculated as the interval between the treatment initiation date (START_DT) and the event occurrence date (EVENT_DT). To ensure data integrity, reports with incomplete, missing, or implausible date entries were excluded from the analysis. TTO distributions were modeled using the Weibull distribution, a flexible parametric approach commonly employed in pharmacovigilance studies. The shape parameter (*β*) of the Weibull model was used to characterize ADE risk patterns over time: *β* < 1 with the 95% confidence interval (CI) upper bound <1 indicated early failure (i.e., AEs tend to occur early during therapy); β ≈ 1 with the 95% CI including 1 indicated random failure (i.e., constant AE risk over time); and β > 1 with the 95% CI lower bound >1 indicated wear-out failure (i.e., increasing AE risk with prolonged treatment duration) (27). At the SOC level, TTO distributions were summarized using medians and interquartile ranges (IQRs). Subgroup comparisons were performed using log-rank tests, and cumulative incidence curves were generated to visualize differential ADE onset patterns across patient subgroups.

### 2.9 Statistical analysis

All statistical analyses and visualizations were performed using R (version 4.2.3). The *p* <0.05 were considered statistically significant. The study design and reporting adhered to the READUS-PV guidelines to ensure methodological transparency and reproducibility (28).

## 3 Results

### 3.1 Baseline characteristics of ADE reports

**Figure 2:**
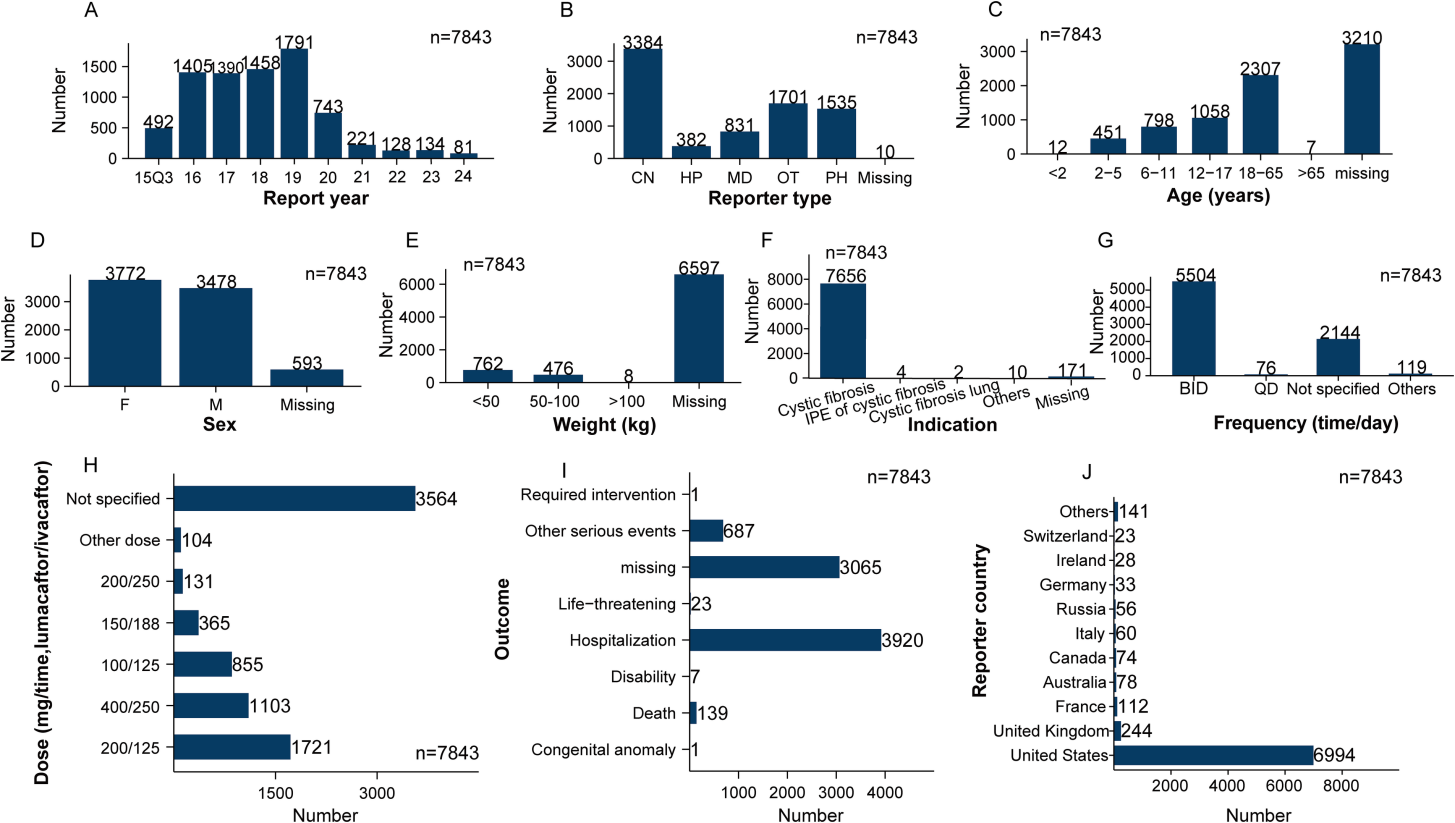
Clinical characteristics of adverse events associated with lumacaftor/ivacaftor reported in FAERS. **(A)** Report year; **(B)** reporter type; **(C)** age; **(D)** sex; **(E)** weight; **(F)** indication; **(G)** frequency (time/day); **(H)** dose (mg/time); **(I)** outcome; **(J)** reported country. CN, consumer; HP, healthprofessional; MD, physician; PH, pharmacist; OT, other health-professional; F, female; M, male.

This study, based on the FAERS database, extracted 15,538,598 reports from Q3 of 2015 to Q4 of 2024. After applying the FDA-recommended deduplication method, 2,221,096 duplicate reports were excluded, resulting in 13,317,502 unique reports for further analysis. By integrating data from the DRUG and REAC files, a total of 7,843 ADEs with LUM/IVA as the PS drug were identified, corresponding to 14,703 adverse event records. A systematic description of the clinical characteristics of these 7,843 reports was performed (Figure 2). In terms of temporal distribution, the number of LUM/IVA-related reports remained high from 2016 to 2019 (with over 1,300 reports per year), but showed a decreasing trend starting since 2019 (Figure 2A). Regarding the type of reporter, consumers were the primary source of reports, accounting for 43.1% (*n* = 3,384), followed by other healthcare professionals (*n* = 1,701, 21.8%), pharmacists (*n* = 1,535, 19.6%), and physicians (*n* = 831, 10.6%) (Figure 2B).

In the 4,633 reports with available age information, the majority were concentrated in the 18–65 years age group (*n* = 2,307, 29.4%), followed by 12–17 years (*n* = 1,058), 6–11 years (*n* = 798), and 2–5 years (*n* = 451) (Figure 2C). In reports with available sex information (7,250 reports), 48.1% were from females (*n* = 3,772) and 44.3% from males (*n* = 3,478), with a close proportion between the two groups (Figure 2D). Notably, more than 80% of the reports lacked weight information (Figure 2E), indicating a significant missing variable in the spontaneous reporting system. Regarding indications, the vast majority of reports explicitly recorded this information (*n* = 7,672, 97.8%), with 97.6% being CF (*n* = 7,565), highly consistent with the approved indication of LUM/IVA (Figure 2F). As for the dosing frequency, 70.2% of the reports indicated a twice-daily regimen (BID, *n* = 5,504), consistent with the recommended dosage in the FDA label. Additionally, 27.3% (*n* = 2,144) of the reports did not provide frequency information (Figure 2G). In terms of dosing, although 45.4% (*n* = 3,564) of the reports lacked detailed dosage data, among those with recorded dosages, the most common were 200/125 mg (*n* = 1,721, 21.9%), 400/250 mg (*n* = 1,103, 14.1%), 100/125 mg (*n* = 855, 10.9%), and 150/188 mg (*n* = 365, 4.7%) (Figure 2H). These dosage distributions align with the FDA-approved drug labeling: 200/125 mg, BID, for patients aged 12 and older; 100/125 mg, twice daily, for children aged 6–11 years; 150/188 mg, twice daily, for children aged 2–5 years with a weight ≥14 kg; and 400/250 mg, which may reflect multiple dose combination reports or regional recording differences.

In terms of adverse event outcomes, the most common event was “hospitalization,” accounting for 50.0% (*n* = 3,920), followed by “other serious events” (8.8%, *n* = 687) (Figure 2I). Notably, 1.8% (*n* = 139) of the reports were related to “death,” indicating potentially serious adverse events that warrant attention. Although clinical trials have shown that LUM/IVA has good tolerability and no clear safety concerns, further systematic research is necessary to establish a causal relationship between these severe events and drug use. Regarding the country of origin for the reports, the majority came from the United States (*n* = 6,994, 89.2%), followed by the United Kingdom (*n* = 244, 3.1%) and France (*n* = 112, 1.4%) (Figure 2I). This distribution may be related to the drug’s market entry timing and its level of usage and accessibility in different countries.

### 3.2 Signals of LUM/IVA and clinical prioritization

To further assess the reliability of adverse event signals, we employed two disproportionality analysis methods to quantify signal strength. At the SOC level, six SOCs showed positive signals in both methods, including infections and infestations [*n* = 3,367, ROR: 5.15 (4.95–5.35), IC025: 2.01], respiratory, thoracic, and mediastinal disorders [*n* = 2,217, ROR: 3.65 (3.49–3.82), IC025: 1.64], procedures and investigations [*n* = 1,495, ROR: 1.83 (1.73–1.93), IC025: 0.72], gastrointestinal disorders [*n* = 1,376, ROR: 1.14 (1.08–1.20), IC025: 0.09], surgical and medical procedures [*n* = 1,273, ROR: 6.47 (6.11–6.85), IC025: 2.50], and congenital, familial, and genetic disorders [*n* = 271, ROR: 6.92 (6.14–7.81), IC025: 2.59]. These findings suggest that these specific SOCs may be associated with increased adverse events in patients treated with LUM/IVA (Table 3).

**Table 3:** Signal strength of lumacaftor/ivacaftor-associated adverse drug event reports at the SOC level.

System organ class	SOC code	Case reports	ROR (95%CI)	IC (IC025)
Infections and infestations	10,021,881	3,367	5.15 (4.95–5.35)	2.07 (2.01)
Respiratory, thoracic, and mediastinal disorders	10,038,738	2,217	3.65 (3.49–3.82)	1.7 (1.64)
General disorders and administration site conditions	10,018,065	1829	0.65 (0.62–0.68)	−0.53 (−0.6)
Investigations	10,022,891	1,495	1.83 (1.73–1.93)	0.8 (0.72)
Gastrointestinal disorders	10,017,947	1,376	1.14 (1.08–1.2)	0.17 (0.09)
Surgical and medical procedures	10,042,613	1,273	6.47 (6.11–6.85)	2.58 (2.5)
Psychiatric disorders	10,037,175	598	0.75 (0.69–0.81)	−0.39 (−0.52)
Nervous system disorders	10,029,205	411	0.34 (0.31–0.37)	−1.49 (−1.64)
Skin and subcutaneous tissue disorders	10,040,785	364	0.42 (0.38–0.46)	−1.21 (−1.36)
Congenital, familial, and genetic disorders	10,010,331	271	6.92 (6.14–7.81)	2.77 (2.59)
Injury, poisoning, and procedural complications	10,022,117	252	0.14 (0.12–0.16)	−2.72 (−2.9)
Metabolism and nutrition disorders	10,027,433	250	0.83 (0.73–0.94)	−0.26 (−0.45)
Musculoskeletal and connective tissue disorders	10,028,395	157	0.2 (0.17–0.23)	−2.28 (−2.52)
Eye disorders	10,015,919	146	0.49 (0.42–0.58)	−1 (−1.24)
Hepatobiliary disorders	10,019,805	142	1.17 (0.99–1.38)	0.22 (−0.02)
Immune system disorders	10,021,428	89	0.5 (0.4–0.61)	−1 (−1.31)
Vascular disorders	10,047,065	88	0.3 (0.25–0.38)	−1.69 (−2)
Reproductive system and breast disorders	10,038,604	86	0.81 (0.66–1.01)	−0.3 (−0.61)
Renal and urinary disorders	10,038,359	72	0.25 (0.2–0.31)	−1.98 (−2.32)
Cardiac disorders	10,007,541	57	0.19 (0.14–0.24)	−2.41 (−2.79)
Social circumstances	10,041,244	44	0.68 (0.5–0.91)	−0.56 (−0.99)
Ear and labyrinth disorders	10,013,993	31	0.48 (0.34–0.68)	−1.05 (−1.56)
Blood and lymphatic system disorders	10,005,329	25	0.1 (0.07–0.15)	−3.29 (−3.85)
Neoplasms benign, malignant, and unspecified (incl cysts and polyps)	10,029,104	24	0.05 (0.04–0.08)	−4.19 (−4.77)
Product issues	10,077,536	21	0.08 (0.05–0.12)	−3.67 (−4.29)
Pregnancy, puerperium, and perinatal conditions	10,036,585	11	0.19 (0.11–0.35)	−2.36 (−3.2)
Endocrine disorders	10,014,698	7	0.18 (0.09–0.38)	−2.45 (−3.47)

Disproportionality analyses were conducted to evaluate signal intensity across different SOC categories. SOC, system organ class; ROR, reporting odds ratio; BCPNN, Bayesian confidence Propagation Neural Network; IC, Information. Component; IC025, 2.5th Percentile of the Information Component. Red numbers indicate values that meet the threshold of the algorithm, representing a positive signal.

After excluding potential interference from the indication and events not directly related to the drug, a total of 221 positive signals were identified (Supplementary Table S1). Among these, 37 signals were consistent with adverse reactions listed in the drug labeling, including cystic fibrosis infectious pulmonary exacerbation [*n* = 1,394, ROR: 1,201.58 (1,126.27–1,281.92), IC025: 9.5], dyspnea [*n* = 517, ROR: 4.00 (3.66–4.37), IC025: 1.83], cough [*n* = 351, ROR: 5.11 (4.59–5.68), IC025: 2.17], infection [*n* = 340, ROR: 9.69 (8.70–10.79), IC025: 3.08], pneumonia [*n* = 224, ROR: 2.92 (2.56–3.33), IC025: 1.34], and rhinitis [*n* = 193, ROR: 4.18 (3.62–4.81), IC025: 1.84]. These were classified as “expected signals.”

When comparing with Kalydeco (ivacaftor) and Symdeko (tezacaftor/ivacaftor) as comparators, no safety signals for ADEs such as anorexia, depression, bloating, aggressive behavior, or suicidal ideation were detected in the former (ivacaftor). In the latter, events like fever, vomiting, bloating, and irritability also did not show positive signals (tezacaftor/ivacaftor) (Supplementary Table S2). Additionally, 61 adverse event signals potentially related to disease progression (disease-expected signals) were identified, with all three drugs meeting the signal threshold, including hospitalization [*n* = 841, ROR: 20.07 (18.71–21.52), IC025: 4.13], pulmonary function test decreased [*n* = 375, ROR: 356.44 (319.64–397.47), IC025: 8.1], malaise [*n* = 215, ROR: 1.99 (1.74–2.27), IC025: 0.78], weight decreased [*n* = 135, ROR: 2.02 (1.71–2.40), IC025: 0.76], sinusitis [*n* = 128, ROR: 5.15 (4.33–6.14), IC025: 2.1], constipation [*n* = 75, ROR: 1.44 (1.15–1.81), IC025: 0.20], and pneumothorax [*n* = 50, ROR: 13.41 (10.15–17.71), IC025: 3.33] (Supplementary Table S1).

**Figure 3:**
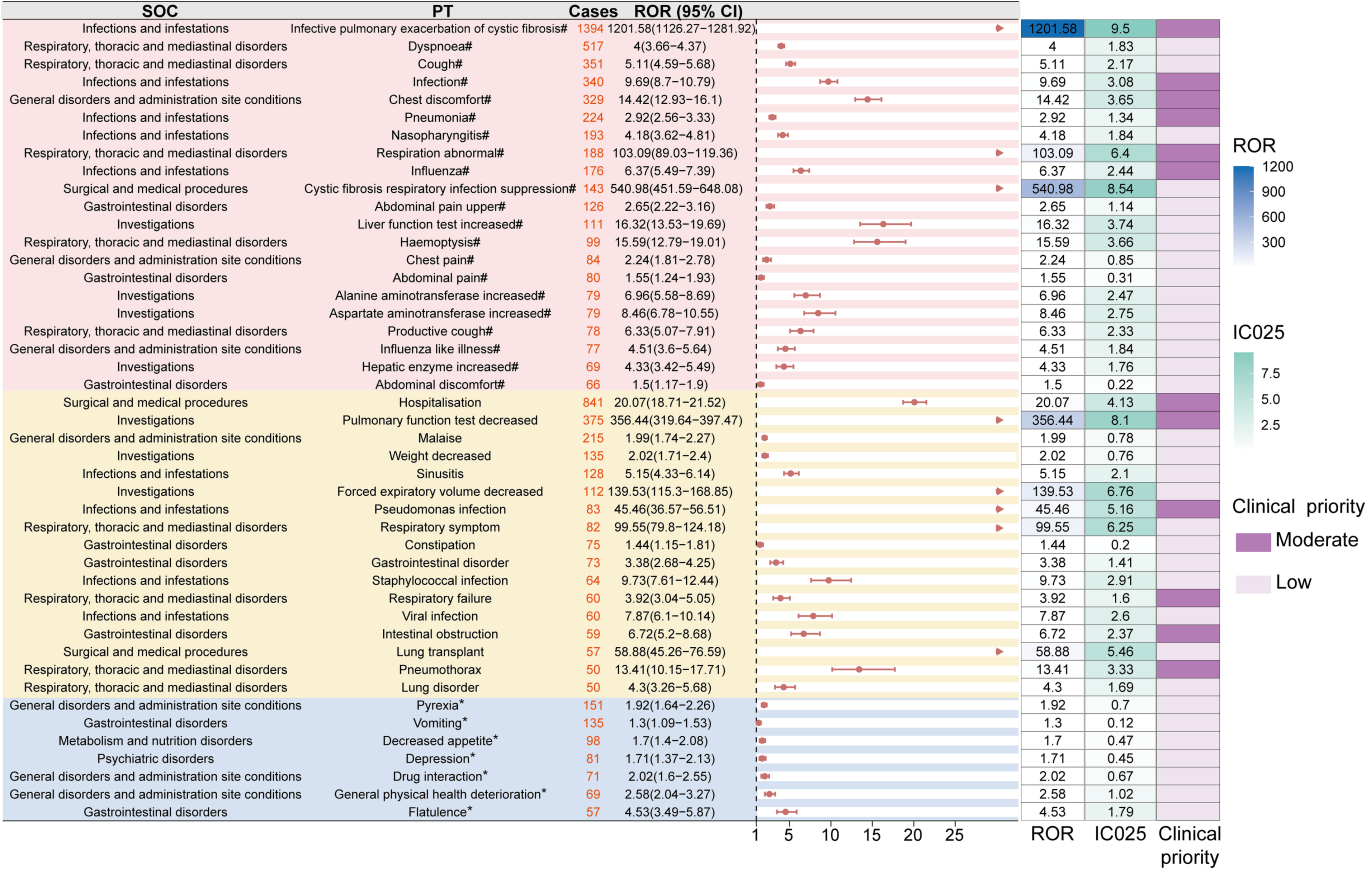
The forest plot displays all positive PTs from the FAERS database with a minimum of 50 reported cases that concurrently meet the signal thresholds of two disproportionality analysis methods. PTs are categorized into “expected signals,” “disease-expected signals,” and “unexpected signals.” Red arrows highlight PTs with a lower bound of the 95% confidence interval for the ROR exceeding 30. The accompanying heatmap on the right illustrates ROR and IC025 values, along with clinical priority scores. Asterisks (*) denote unexpected signals, while hash symbols (#) indicate disease-expected signals. Dark purple represents moderate clinical priority, and light purple indicates low clinical priority. SOC, system organ class; PT, preferred term; ROR, reporting odds ratio; IC025, information component 2.5th percentile.

Notably, we also identified 123 “unexpected signals” not listed in the drug label, including pyrexia [*n* = 151, ROR: 1.92 (1.64–2.26), IC025: 0.70], vomiting [*n* = 135, ROR: 1.30 (1.09–1.53), IC025: 0.12], decreased appetite [*n* = 98, ROR: 1.70 (1.40–2.08), IC025: 0.47], depression [*n* = 81, ROR: 1.71 (1.37–2.13), IC025: 0.45], flatulence [*n* = 57, ROR: 4.53 (3.49–5.87), IC025: 1.79], aggression [*n* = 32, ROR: 3.38 (2.39–4.78), IC025: 1.25], suicidal ideation [*n* = 31, ROR: 1.63 (1.15–2.32), IC025: 0.20], mood altered [*n* = 25, ROR: 4.21 (2.84–6.24), IC025: 1.50], hypoglycemia [*n* = 20, ROR: 2.02 (1.31–3.14), IC025: 0.39], pancreatitis [*n* = 18, ROR: 1.84 (1.16–2.92), IC025: 0.21], steatorrhea [*n* = 17, ROR: 42.73 (26.45–69.02), IC025: 4.71], and rhabdomyolysis [*n* = 17, ROR: 2.16 (1.34–3.47), IC025: 0.43], among others. To enhance the clinical applicability of adverse event signals, we introduced a semi-quantitative scoring system to classify the clinical priority of each PT. Ultimately, we identified 189 low-priority signals, 32 medium-priority signals, and no high-priority signals. The medium-priority PTs included infective pulmonary exacerbation of CF, hospitalization, pulmonary function test decreased, infection, chest discomfort, pneumonia, influenza, intestinal obstruction, and distal intestinal obstruction syndrome. Common ADEs such as dyspnea, cough, malaise, nasopharyngitis, pyrexia, weight decreased, vomiting, and sinusitis were classified as low priority (Supplementary Table S1). To visually present these results, we used forest plots and heat maps to display the ROR values, IC025 values, and clinical priority levels for all positive signals with at least 50 cases (Figure 3).

### 3.3 Signal differences of LUM/IVA

**Figure 4:**
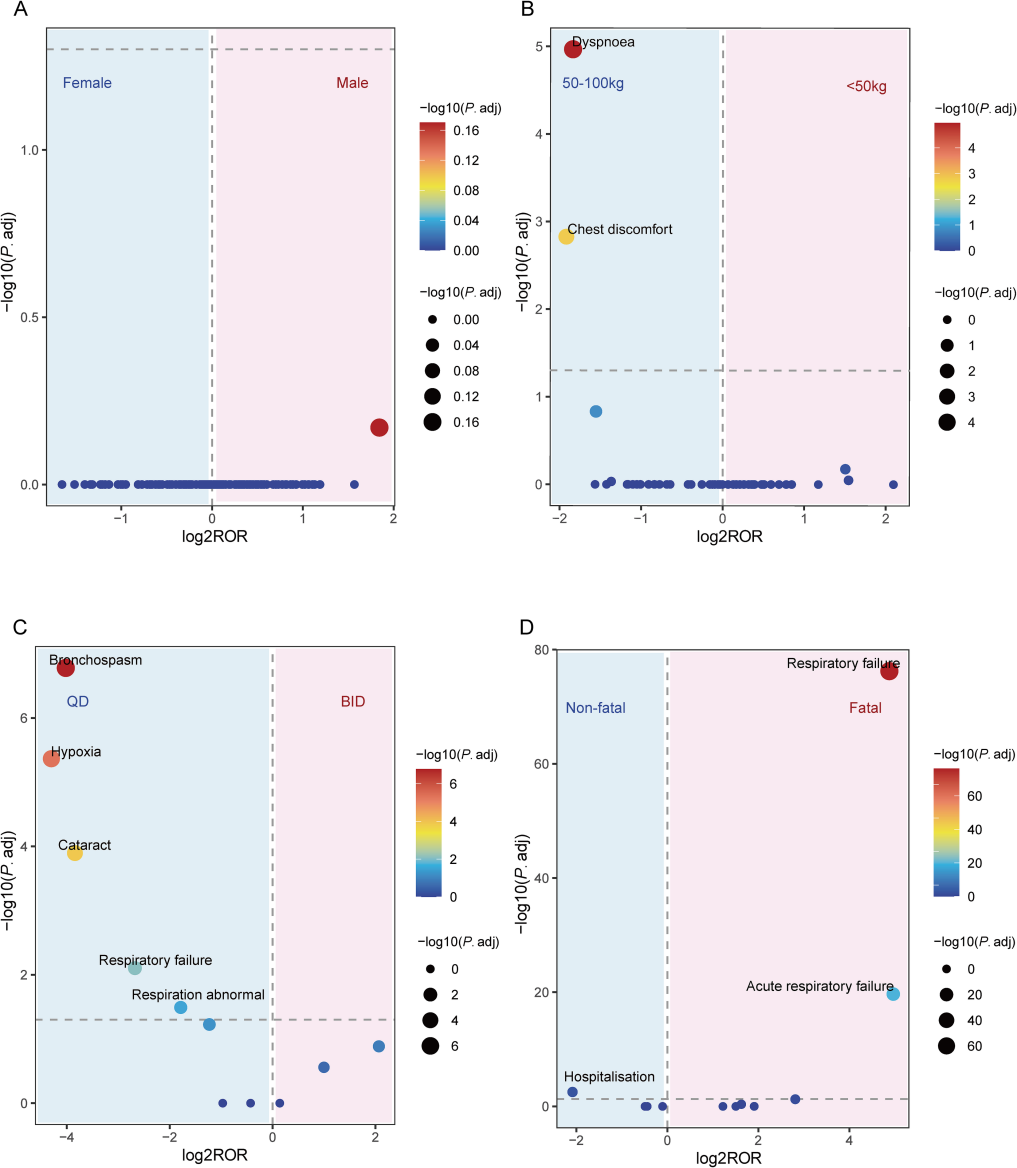
Volcano plots illustrating differential signal detection associated with lumacaftor/ivacaftor. **(A)** Sex-based differences in adverse drug events reporting between female and male patients. **(B)** Weight-based differences comparing patients weighing 50–100 kg versus those under 50 kg. **(C)** Signal variation based on dosing frequency: once daily (QD) versus twice daily (BID). **(D)** Differences in signal strength between fatal and non-fatal case reports. The X-axis represents the log_2_-transformed reporting odds ratio (log_2_ROR), calculated using the ROR method. The Y-axis displays the negative base-10 logarithm of the adjusted *p*-value (-log_10_P.adj), derived from the Chi-square test. Each dot represents a preferred term, with its color and size proportional to its -log_10_P.adj value. Statistically significant associations (*P*.adj < 0.05) are indicated by greater absolute log_2_ROR values, suggesting stronger disproportionality between subgroups.

Figure 4 shows a volcano plot illustrating the differences in LUM/IVA-related signals based on variables such as sex, weight, dosing frequency, and whether the report involved a fatal outcome. In the sex-related subgroup analysis, no significant difference in signals was observed (Figure 4A). In the weight subgroup, compared to patients weighing less than 50 kg, risk signals were “chest discomfort” (*P*.adj = 0.001, ROR = 0.26, 95% CI: 0.14–0.50) and “dyspnea” (*P*.adj = 1.08 × 10^−5^, ROR = 0.28, 95% CI: 0.17–0.46) in patients weighing 50–100 kg (Figure 4B). It should be noted that weight-stratified analyses were conducted based on reports with available weight information only, given that more than 80% of FAERS reports lacked documented body weight. In the dosing frequency analysis, compared to those receiving BID regimen, patients on QD regimen were more likely to experience the following adverse events: abnormal respiration (*P*.adj = 0.03, ROR = 0.29, 95% CI: 0.13–0.63), respiratory failure (*P*.adj = 0.01, ROR = 0.16, 95% CI: 0.05–0.45), bronchospasm (*P*.adj = 1.66 × 10^−7^, ROR = 0.06, 95% CI: 0.02–0.19), cataract (*P*.adj = 1.3 × 10^−4^, ROR = 0.07, 95% CI: 0.02–0.25), and hypoxia (*P*.adj = 4.31 × 10^−6^, ROR = 0.05, 95% CI: 0.01–0.19) (Figure 4C). In the fatal report analysis, significant high-risk signals identified included respiratory failure (fatal vs. non-fatal: *P*.adj = 5.91 × 10^−77^, ROR = 29.40, 95% CI: 17.36–49.78) and acute respiratory failure (*P*.adj = 2.14 × 10^−20^, ROR = 31.20, 95% CI: 11.23–86.70). Additionally, among non-fatal adverse events, hospitalization also showed a significant signal (*P*.adj = 0.003, ROR = 0.24, 95% CI: 0.10–0.53) (Figure 4D).

### 3.4 Descriptive analysis of concomitant drugs

**Figure 5:**
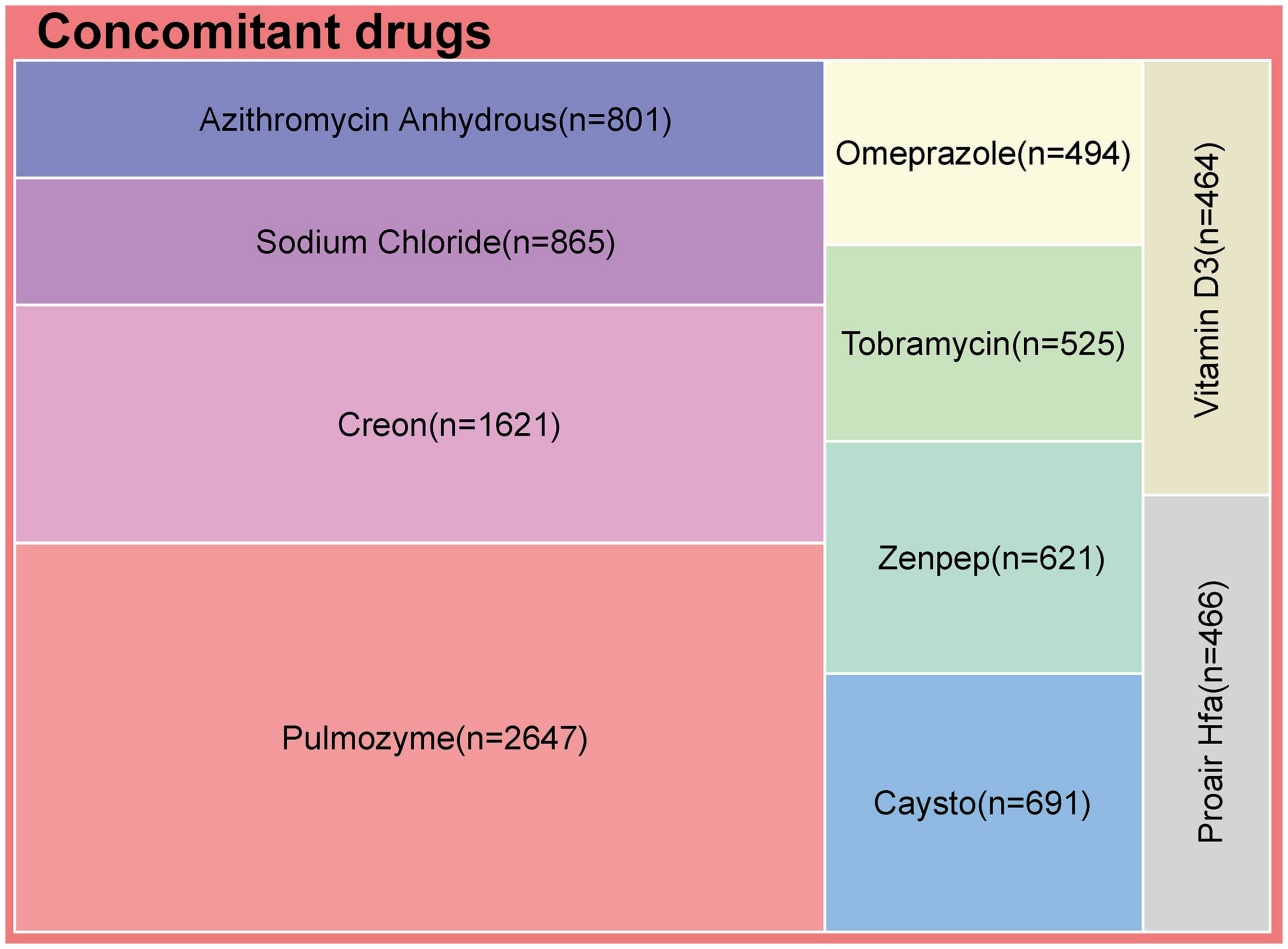
The 10 most commonly reported concomitant drugs in adverse event reports associated with lumacaftor/ivacaftor.

In the 7,843 LUM/IVA-related ADEs, a total of 4,423 reports involved concomitant drug use, covering more than 1,000 different medications. Figure 5 shows the top 10 most frequently reported concomitant drugs. Among these 4,423 reports, the most commonly co-administered drug with LUM/IVA was pulmozyme, with a total of 2,647 cases. Other frequently reported concomitant drugs included creon (1,621 cases), sodium chloride (865 cases), azithromycin anhydrous (801 cases), cayston (691 cases), zenpep (621 cases), tobramycin (525 cases), omeprazole (494 cases), proair HFA (466 cases), and vitamin D3 (464 cases).

### 3.5 Sensitivity analysis

To exclude the potential confounding effects of concomitant drug use, this study excluded all cases involving the use of other medications and only included 3,420 ADEs related to the sole use of LUM/IVA for analysis. Based on this, a disproportionality analysis identified 141 positive signals. Notably, some of these signals remained robust in the sensitivity analysis, including infective pulmonary exacerbation of cystic fibrosis, hospitalization, dyspnea, cough, malaise, infection, pneumonia, nasopharyngitis, pyrexia, weight decreased, depression, aggression, mood altered, pancreatitis, hypoglycemia, and rhabdomyolysis (see Supplementary Table S3). Overall, the sensitivity analysis results were consistent with the trends observed in the primary analysis, further confirming the stability of these signals and supporting a potential association between these events and LUM/IVA use.

### 3.6 Logistic regression analysis

Previous studies have suggested that LUM/IVA treatment may be associated with an increased risk of depression, particularly in female adolescents with CF (29). To further assess the risk of depression associated with LUM/IVA under the influence of various confounding factors, this study employed both univariate and multivariate logistic regression analyses. After excluding missing data, a total of 3,440 complete ADEs were included, which contained key information such as age, sex, reporter type, reporting country, dosing frequency, and whether concomitant medication was used. The results of the univariate logistic regression analysis showed that, compared to females, males were a significant protective factor, significantly reducing the risk of LUM/IVA-related depression (OR = 0.226, 95% CI: 0.086–0.597, *p* = 0.003). Reports submitted by consumers were more likely to report depressive events compared to those submitted by healthcare professionals, though the result did not reach statistical significance (OR = 2.062, 95% CI: 0.972–4.372, *p* = 0.059). No significant associations were observed with variables such as age, report country, dosing frequency, or concomitant medication use. The multivariate logistic regression analysis results were consistent with those of the univariate analysis (see Table 4), further confirming that male sex was an independent protective factor compared to female sex (OR = 0.224, 95% CI: 0.085–0.590, *p* = 0.002).

**Table 4:** Univariate and multivariate logistic regression analysis of risk factors for lumacaftor/ivacaftor-associated depression.

Characteristics	Total(N)	Univariate analysis		Multivariate analysis	
		Odds ratio (95% CI)	*p-*value	Odds ratio (95% CI)	*P-*value
Age (years)	3,440				
18–65	1,658	Reference		–	–
<18	1,782	1.243 (0.586–2.635)	0.571	–	–
Sex	3,440				
Female	1,764	Reference		Reference	
Male	1,676	0.226 (0.086–0.597)	**0.003**	0.224 (0.085–0.590)	**0.002**
Reporter type	3,440				
Health professionals	2,084	Reference		Reference	
Consumer	1,356	2.062 (0.972–4.372)	0.059	2.106 (0.992–4.472)	0.053
Report country	3,440				
United States	3,105	Reference		–	–
Others	335	2.030 (0.767–5.376)	0.154	–	–
Frequency	3,440				
BID	3,378	Reference		–	–
QD	62	2.035 (0.272–15.214)	0.489	–	–
Mono-therapy	3,440				
No	2,510	Reference		–	–
Yes	930	1.080 (0.474–2.461)	0.854	–	–

Statistically significant values (*P* < 0.05) are shown in bold.

### 3.7 Time to onset analysis at the overall perspective

**Figure 6:**
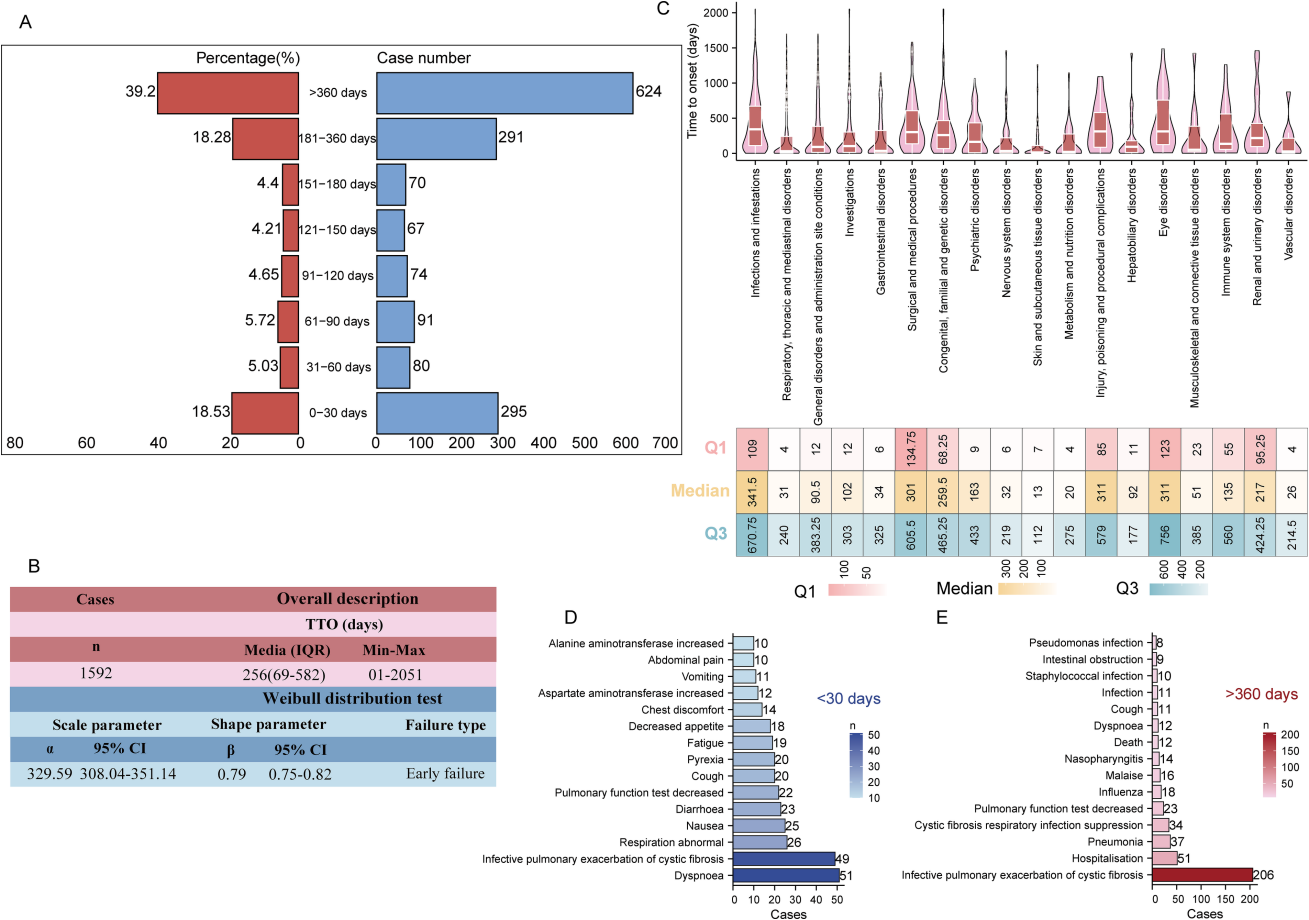
Comprehensive time-to-onset (TTO) analysis. **(A)** Butterfly plot illustrating the distribution of TTO reports across predefined time intervals. The right panel displays the absolute number of reports, while the left panel indicates the corresponding percentage. **(B)** Descriptive summary including the median, extreme values, and results of the Weibull distribution test for all TTO reports. **(C)** Box plot depicting the TTO distribution for ADEs categorized by SOC. The white line within each box denotes the median TTO, with the lower and upper edges representing Q1 and Q3, respectively. **(D)** Bar chart showing the top 15 PTs with TTOs less than 30 days, ranked by the number of reported cases. **(E)** Bar chart showing the top 15 PTs with TTOs greater than 360 days, similarly ranked by case frequency. ADE, adverse drug events; SOC, system organ class; Q1, first quartile; Q3, third quartile; PT, preferred term.

This study included 1,592 valid reports for TTO analysis. The butterfly plot in Figure 6A shows the distribution and proportions of ADE onset times. The results revealed that most ADEs occurred after the 360 days of treatment initiation (*n* = 624, 39.2%), within the first month (*n* = 295, 18.53%), and within the second month (*n* = 80, 5.03%). Figure 6B presents the statistical characteristics of the overall TTO. The median onset time for all adverse events was 256 days (interquartile range: 69–582 days). The Weibull distribution test indicated that the event occurrence pattern follows an “early failure” type, with a shape parameter *β* of 0.79 (95% CI: 0.75–0.82), suggesting that the probability of most adverse events occurring decreases over time. At the SOC level, TTO analysis covered 18 SOC categories (Figure 6C). SOCs with a median TTO of less than 30 days included: skin and subcutaneous tissue disorders (13 days), metabolic and nutritional disorders (20 days), and vascular disorders (26 days). In contrast, SOCs with a median TTO exceeding 180 days included: infections and infestations (341.5 days), surgical and medical procedure (301 days), congenital, familial, and genetic disorders (259.5 days), injury, poisoning, and procedural complications (311 days), eye disorders (311 days), and renal and urinary disorders (217 days). To optimize the monitoring and management of adverse events, the study further analyzed reports with a TTO of less than 30 days and more than 360 days, listing the top 15 most frequently reported PT for each. In events with a TTO of less than 30 days, the five most common PTs were: dyspnea (*n* = 51), infective pulmonary exacerbation of CF (*n* = 49), abnormal respiration (*n* = 26), nausea (*n* = 25), and diarrhea (*n* = 23) (Figure 6D). In reports with a TTO greater than 360 days, the top five PTs were: infective pulmonary exacerbation of CF (*n* = 206), hospitalization (*n* = 51), pneumonia (*n* = 37), CF respiratory infection suppression (*n* = 34), and (*n* = 23) (Figure 6E).

### 3.8 Stratified analysis of TTO

**Figure 7:**
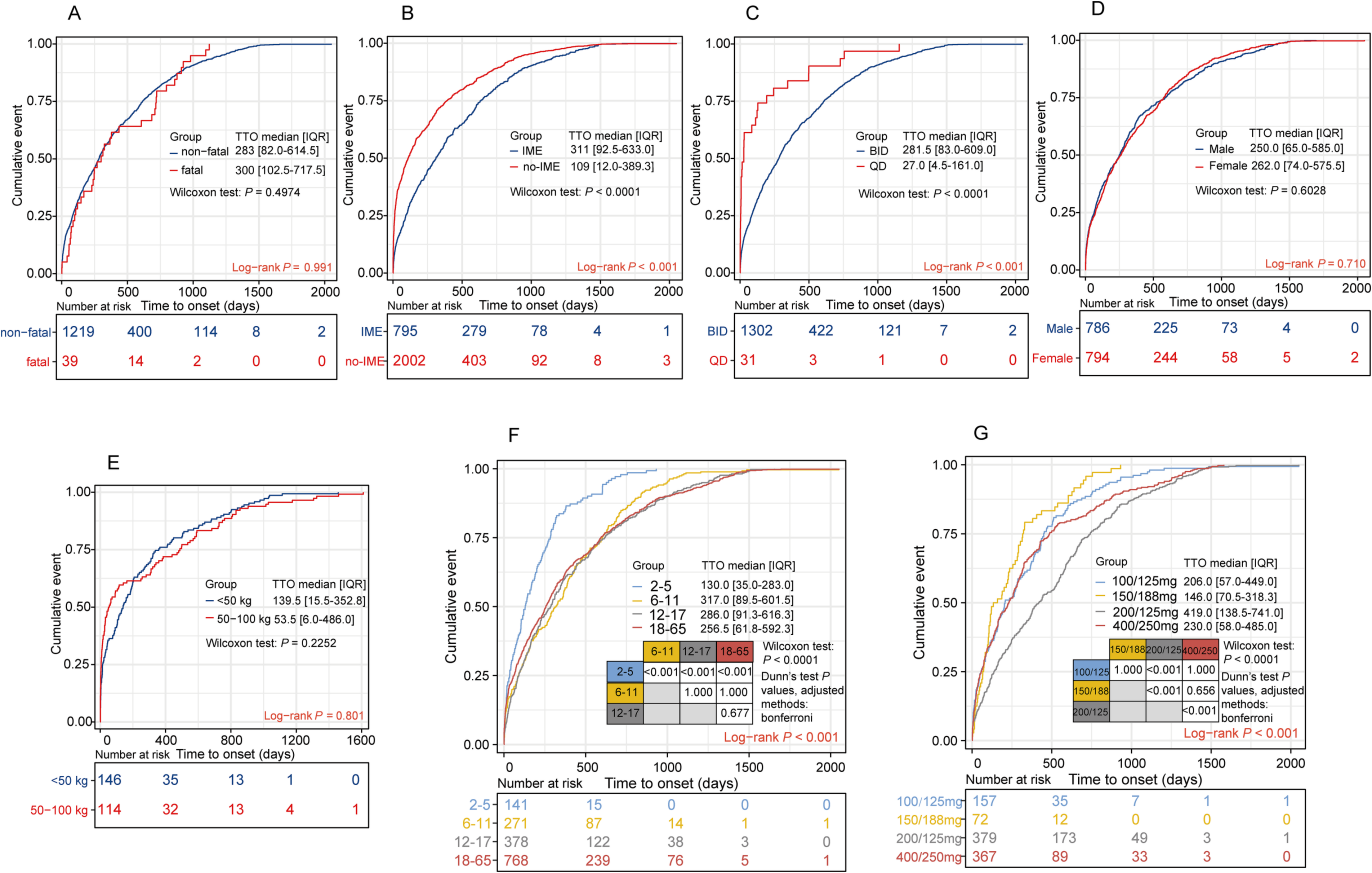
Cumulative incidence analysis of time to onset (TTO) in lumacaftor/ivacaftor-associated adverse event reports across subgroups. Panels depict TTO differences across the following subgroups: **(A)** Fatal vs. non-fatal outcomes, **(B)** important medical events (IME) vs. non-IME, **(C)** once-daily (QD) vs. twice-daily (BID) dosing, **(D)** sex, **(E)** weight categories, **(F)** age groups, and **(G)** dosage levels. Statistical comparisons were performed using the non-parametric Wilcoxon rank sum test. TTO, time to onset; IQR, interquartile range; IME, important medical events; DME, designated medical events.

To further explore the potential factors influencing the TTO of LUM/IVA-related adverse events, we conducted a stratified analysis of TTO controlling for variables such as sex, age, weight, dosage, dosing frequency, fatal reports, and Important Medical Events (IME). In subgroups such as fatal reports (Figure 7A) (log-rank *p* = 0.991; Wilcoxon *p* = 0.500), sex (Figure 7D) (log-rank *p* = 0.710; Wilcoxon *p* = 0.603), and weight (Figure 7E) (log-rank *p* = 0.801; Wilcoxon *p* = 0.225), no significant differences in TTO were observed. However, compared to IME-related reports [median TTO (IQR): 311 days (93–633)], the TTO of non-IME reports was significantly shorter [median TTO (IQR): 109 days (12–389)] (Figure 7B) (log-rank *p* < 0.001; Wilcoxon *p* < 0.0001), indicating that IME-related events occurred later. In terms of dosing frequency, the QD group had a significantly later TTO than the BID group [QD group median TTO (IQR): 282 days (83–609); BID group median TTO (IQR): 27 days (5–161)] (Figure 7C) (log-rank *p* < 0.001; Wilcoxon *p* < 0.0001).

The cumulative incidence also differentiated among age subgroups (log-rank *p* < 0.001) (Figure 7F). The median onset time of adverse events in children aged 2–5 years was significantly later than in the 6–11 years [130 days (35–283) vs. 317 days (90–602), Wilcoxon *p* < 0.001], 12–17 years [130 days (35–283) vs. 286 days (91–616), Wilcoxon *p* < 0.001], and 18–65 years groups [130 days (35–283) vs. 257 days (62–592), Wilcoxon *p* < 0.001]. However, no significant differences were found between the 6–11 years, 12–17 years, and 18–65 years groups. Notably, the dosage subgroup also showed significant TTO differences (log-rank *p* < 0.001; Wilcoxon *p* < 0.0001) (Figure 7G). Compared to the 200/125 mg dose group, the 100/125 mg group [median TTO (IQR): 419 days (139–741) vs. 206 days (57–449), Dunn’s test *p* < 0.001], 400/250 mg group [419 days (139–741) vs. 230 days (58–485), Dunn’s test *p* < 0.001], and 150/188 mg group [419 days (139–741) vs. 146 days (71–318), Dunn’s test *p* < 0.001] all exhibited earlier onset times for adverse events (Figure 7G). This study found that age, dosing frequency, dosage, and IME-related factors significantly influence the onset time of adverse events, which can help optimize personalized treatment and risk management strategies.

## 4 Discussion

We conducted an in-depth analysis of post-marketing pharmacovigilance data for LUM/IVA, identifying both known ADEs listed in the drug labeling and previously unreported or relatively rare ADEs. This analysis provides the first systematic overview of adverse event reports related to LUM/IVA in the FAERS database. Annual ADE reports for LUM/IVA showed a significant decline in 2020, which may be attributed to the introduction of elexacaftor/tezacaftor/ivacaftor (ELX/TEZ/IVA), as the use of the latter likely led to a reduction in LUM/IVA usage. Data from the UK Cystic Fibrosis Registry in 2022 indicated that from January 2021 to December 2022, the number of patients treated with ELX/TEZ/IVA increased from 4,195 to 6,846, while the use of LUM/IVA decreased during this period. Additionally, drug-related ADEs may be influenced by the indication. In this study, 97.6% of the ADE reports were from CF patients, which enhanced the sensitivity of our analysis. Geographically, the United States accounted for the largest proportion of reports (89.2%). This difference may stem from the fact that the U. S. was the first country to approve the use of LUM/IVA in 2015, facilitating its early and widespread adoption.

### 4.1 Known and unexpected signals

Our comprehensive pharmacovigilance analysis identified 221 positive signals related to LUM/IVA across 18 different SOC categories. Among these positive signals, the most commonly reported ADEs included infective pulmonary exacerbation of CF (1,394 cases), hospitalization (841 cases), dyspnea (517 cases), decline in pulmonary function testing (375 cases), cough (351 cases), and infection (340 cases). Additionally, we identified signals consistent with those listed in the drug label, such as dyspnea (517 cases), influenza (176 cases), abdominal pain upper (126 cases), hemoptysis (99 cases), chest pain (84 cases), and abdominal pain (80 cases), further validating the reliability of our analysis. A multicenter observational study indicated that respiratory-related adverse events (48.1%) were the main cause of LUM/IVA discontinuation, with dyspnea/chest tightness accounting for 24.7% (30). Furthermore, two phase III, randomized, double-blind, placebo-controlled studies in CF patients aged 12 and older revealed that most of the reported adverse events in the LUM/IVA group were respiratory-related, with the majority being mild to moderate in severity, including dyspnea (13.0% vs. 7.8%) and chest tightness (8.7% vs. 5.9%) (7). Another study, the PROGRESS study, found that the most common ADEs included infective pulmonary exacerbation, cough, increased sputum, and hemoptysis (31). Additionally, a case study reported a 25-year-old female CF patient who experienced a recurrence of hemoptysis during menstruation while receiving LUM/IVA treatment, although the final analysis suggested that LUM interfered with the effectiveness of hormone therapy (32). Hemoptysis is a common complication in CF patients, with approximately 9% experiencing this symptom within 5 years (32). Since hemoptysis is a clinical emergency, we need to remain vigilant for potential ADEs in addition to disease progression.

We also identified unexpected ADEs related to LUM/IVA, including suicidal ideation [*n* = 31, ROR: 1.63 (1.15–2.32), IC025: 0.20], hypoglycemia [*n* = 20, ROR: 2.02 (1.31–3.14), IC025: 0.39], rash popular [*n* = 13, ROR: 2.45 (1.42–4.21), IC025: 0.52], and depression [*n* = 81, ROR: 1.71 (1.37–2.13), IC025: 0.45]. Suicidal ideation can lead to fatal outcomes, causing significant emotional distress for families and substantial economic burden on society (33). A previous systematic review suggested that LUM/IVA may be associated with drug-induced suicide, which is not mentioned in the drug labeling (34). Case reports have documented five adolescent female CF patients who experienced exacerbation of anxiety and depression after starting LUM/IVA combination therapy, leading two of them to develop suicidal ideation, while the other three attempted suicide and required psychiatric hospitalization (29). Another case report and disproportionality analysis also suggested a potential risk of suicidal ideation associated with LUM/IVA (35). While the specific mechanism of suicidal ideation induced by LUM/IVA remains unclear, it is essential to include this significant severe ADE in the drug labeling as a warning. Hypoglycemia is defined as abnormally low plasma glucose levels, which can potentially harm patients. A retrospective study found that one patient using LUM/IVA experienced persistent hypoglycemia (36). Two studies investigating the effects of CFTR on *β*-cells and pancreatic function suggested that CFTR plays a critical role in pancreatic function and β-cell activity, significantly impacting insulin secretion (37, 38). Therefore, when using LUM/IVA in patients with CF and diabetes, it is essential to monitor peripheral blood glucose levels and remain vigilant for the onset of hypoglycemia. Rashes are among the most common idiosyncratic adverse events following the initiation or re-exposure to CFTR modulator therapy. Two patients developed rashes after using LUM/IVA (39). A case report also described an 11-year-and-9-month-old girl with CF (F508del homozygous), who developed pruritic rashes and urticaria 6 days after starting LUM/IVA (40). The rash resolved successfully after a 15-min interval and a rapid increase in the dose of LUM/IVA to desensitize the patient. Our findings provide a detailed catalog for clinical use of LUM/IVA, helping clinicians to identify unlisted or rare ADEs early in clinical practice.

In an international study covering nine countries and involving more than 6,000 adolescents and adults with CF, approximately 25% of the U. S. sample self-reported an increase in depressive symptoms (41). Although previous research has shown that ivacaftor demonstrates sustained central nervous system activity, particularly its affinity for 5-HT2 receptor subtypes, which may significantly improve the quality of life and increase well-being for CF patients, including those with depression and/or anxiety, the study also found that five adolescent CF females developed new or worsened depressive or anxiety symptoms after starting LUM/IVA combination therapy (42). In most cases, these symptoms improved after discontinuing the medication. Another study indicated a temporal association between ELX/TEZ/IVA treatment and psychiatric ADEs, but it was unable to clarify which component of the combination therapy caused the neuropsychiatric effects or whether the impact was due to a single ingredient (43). Therefore, further research into drug-induced psychiatric ADEs related to the drug itself or its components remains necessary. Exploring potential biological mechanisms is crucial for ensuring the safe use of medications and for the early identification of potential neuropsychiatric ADEs (44). This study, through univariate and multivariate logistic regression analysis, aimed to further clarify relevant risk factors and sex differences. The results showed that the risk of depression in male patients was significantly lower compared to female patients. This finding suggests that sex differences may play an important role in the mental health issues triggered by drug therapy. Previous studies have indicated that 3.1% of males and 4.6% of females report experiencing “moderate/severe” depression (45), a finding that aligns with our results. First, LUM/IVA may influence CFTR expression in the brain, thereby interfering with neural function and potentially causing or exacerbating depressive symptoms (46). Secondly, women may be more susceptible to emotional distress during treatment due to hormonal fluctuations and more complex social and psychological pressures, leading to a worsening of depressive symptoms. Additionally, the quality of life for female CF patients is generally lower than that of males (47), and this disparity may exacerbate their emotional issues, making psychiatric disorders more likely to occur in women. Multivariate analysis further confirmed the crucial role of male sex as an independent protective factor in the risk of depression. Therefore, clinical practice should focus on sex and individual differences, regularly assess mental health status, and develop personalized treatment plans based on the patient’s specific circumstances to mitigate the adverse effects that drug therapy might have on depressive symptoms. To evaluate whether these ADEs are related to CF, we compared patients receiving LUM/IVA with those treated with Kalydeco (ivacaftor) and Symdeko (tezacaftor/ivacaftor). The safety signals commonly observed in LUM/IVA, ivacaftor, and LUM/IVA treatments suggest that these ADEs may be related to CF itself rather than being specific to LUM/IVA. However, adverse events such as aggressive behavior, suicidal ideation, mood changes, hypoglycemia, and rhabdomyolysis [*n* = 17, ROR: 2.16 (1.34–3.47), IC025: 0.43] were considered specific to LUM/IVA. These results further enhance the specificity and stability of our study.

### 4.2 Subgroup analysis

The volcano plot analysis showed that no significant differential signals were observed in the sex-related subgroup, suggesting that sex may have a limited impact on adverse events. However, in the weight subgroup analysis, patients weighing between 50 and 100 kg were more likely to report “chest discomfort” and “dyspnea” compared to those weighing less than 50 kg. This may be related to the increased pulmonary burden in patients with higher body weight (48). However, the interpretation of weight-stratified signals should be approached with caution. Given the substantial proportion of missing body weight information in FAERS reports, the subgroup analysis was limited to a selected subset of cases with complete data. As reporting of weight is not mandatory in spontaneous reporting systems, this subset may not be fully representative of the overall population receiving LUM/IVA, potentially introducing selection bias. In the dosing frequency analysis, the results indicated that patients receiving QD dosing were more likely to experience adverse reactions such as abnormal respiration, respiratory failure, bronchospasm, cataract, and hypoxia compared to those receiving BID dosing. This finding may be related to the underlying pathological conditions and disease progression of the patients, where once-daily dosing may result in insufficient therapeutic concentrations of the drug. The fatal report analysis revealed that respiratory failure and acute respiratory failure were significant high-risk signals (Figure 4D), suggesting that in LUM/IVA treatment, patients with more comorbidities may be more likely to experience adverse outcomes, leading to this bias. This study highlights the important roles of weight, dosing frequency, and fatal reports in LUM/IVA-related ADEs, emphasizing that clinical practice should involve personalized treatment plans based on the patient’s weight and dosing frequency to reduce the risk of severe adverse reactions during treatment.

### 4.3 TTO analysis

The side effects of LUM/IVA therapy were more concentrated in the early and late stages of treatment. The TTO analysis further revealed differences between various SOC categories. The median TTO for skin, metabolic and nutritional disorders, and vascular diseases was less than 30 days, suggesting that these adverse events typically occur early in treatment. In contrast, SOCs related to infections, surgical procedures, and eye disorders had longer TTOs, with medians exceeding 180 days. Notably, the TTO for infections and infestations was as long as 341.5 days, indicating that these adverse events are more likely to result from the cumulative effects of long-term treatment or from ongoing immune system stress during extended therapy. LUM/IVA may affect airway epithelial cell function by modulating the CFTR channel, potentially leading to long-term immune dysfunction (49, 50). A deeper analysis of reports with TTOs of less than 30 days and over 360 days revealed that the most common ADEs in the early onset group included dyspnea, infective pulmonary exacerbation of cystic fibrosis, abnormal respiration, nausea, and diarrhea. These reactions are typically linked to the drug’s direct impact on the respiratory system and gastrointestinal side effects. Respiratory symptoms might be associated with the drug’s irritant effects on the airways and the resulting pulmonary inflammation, while gastrointestinal symptoms could be related to the drug’s effects on gut microbiota or changes in gastrointestinal function. Additionally, long-term use of these drugs may impact the gut microbiome, contributing to gastrointestinal discomfort and infection responses (51). Therefore, clinical attention should be particularly focused on these early and long-term adverse reactions, especially respiratory-related complications, to allow timely intervention and optimize treatment strategies, ensuring patient safety during LUM/IVA therapy. This study also explored various potential factors influencing the TTO of LUM/IVA-related ADEs and found that age and IME significantly affected TTO. The TTO for children aged 2–5 years was notably earlier compared to other age groups, which may be due to the immature metabolic enzyme system in children, a more sensitive immune system, and the impact of body weight on drug dosage (52, 53). Given that younger patients have higher metabolic rates and metabolize drugs faster, adverse events occur earlier, whereas the latent period in adult patients may be longer (54). Meanwhile, the presence of IME significantly delayed the occurrence of TTO, which could be related to immune system abnormalities, inflammatory responses, and drug interactions. Patients with IME have slower drug metabolism, leading to prolonged drug accumulation in the body, thereby delaying the onset of adverse events. Our findings provide valuable insights for optimizing personalized treatment and risk management. However, given the lower reporting rate of TTO, these findings should be interpreted with caution.

### 4.4 Limitations

The limitations of this study primarily lie in the following aspects. First, our analysis largely relies on spontaneous reporting databases, which are susceptible to selection bias from reporters (55). Since these systems depend on the voluntary reporting of adverse events by patients, doctors, and healthcare providers, there is a potential underreporting or incomplete reporting of cases, which could lead to an underestimation of certain adverse events. Although the FAERS data provide valuable insights into the use of LUM/IVA, these data lack standardization in terms of patient disease progression, comorbidities, and treatment regimens, which could affect the comparability of results. Second, this study did not account for all potential confounding factors (56). While we explored the impact of variables such as sex, weight, and dosing frequency through subgroup analysis, other factors, such as lifestyle, prior treatment history, or concomitant medications, may also influence the incidence and type of adverse events. These factors were not fully controlled in this study. In addition, a substantial proportion of reports lacked key covariates such as body weight and dosing frequency, which limited the sample size available for stratified analyses. Although this limitation is inherent to spontaneous reporting databases, it may restrict the generalizability of subgroup-specific findings. Therefore, results derived from weight- and frequency-stratified analyses should be considered exploratory and hypothesis-generating rather than confirmatory. Another important consideration is reporting bias inherent to spontaneous reporting systems. In our study, the majority of reports originated from the United States and were submitted by consumers. This pattern likely reflects differences in regional pharmacovigilance infrastructure, public awareness, and reporting culture rather than true geographic variation in drug safety. Consumer-submitted reports, while valuable for signal detection, may be influenced by subjective symptom perception and media attention, potentially contributing to stimulated or notoriety bias. Therefore, the observed reporting patterns should not be interpreted as global incidence estimates or definitive safety trends across regions. Additionally, although we conducted an in-depth exploration of the long-term use of LUM/IVA and its potential adverse effects, the long-term follow-up data on drug use were relatively limited. Some datasets had shorter follow-up periods, which may not capture all long-term adverse events, particularly for elderly patient populations. The potential risks after prolonged use are still not fully understood. Finally, despite our efforts to use TTO as a core metric in the analysis, the reporting rate of TTO was low, leading to an insufficient sample size that could limit the robustness of our conclusions. Therefore, further large-scale, prospective, randomized controlled studies will be beneficial to validate the findings of this study and provide more in-depth evidence on the long-term safety of LUM/IVA. Overall, while this study provides important insights into the adverse effects and associated risks of LUM/IVA treatment, these limitations highlight the need for future research to more systematically and comprehensively assess the safety of the drug, particularly its long-term effects in different patient populations.

## 5 Conclusion

This study presents a comprehensive post-marketing safety evaluation of LUM/IVA based on data from the FAERS spanning 2015 to 2024. Consistent with clinical trial findings and the current drug label, our analysis confirmed known safety signals such as respiratory adverse events and pulmonary exacerbations. Importantly, we also identified previously unreported and rare adverse events—including suicidal ideation, depression, and hypoglycemia—that are not currently included in the prescribing information. Logistic regression analysis further revealed potential risk factors associated with LUM/IVA-related depression, while the TTO analysis characterized the temporal patterns and subgroup differences in cumulative adverse event occurrence. These findings highlight the necessity for strengthened pharmacovigilance, particularly with respect to psychiatric symptoms and long-term adverse outcomes, and support consideration for label updates to reflect emerging safety concerns.

## Data Availability

The original contributions presented in the study are included in the article/Supplementary material, further inquiries can be directed to the corresponding author.

## Supplementary material

Supplementary Table S1 Comprehensive results of 221 positive signals identified by meeting the threshold criteria of both disproportionality analysis algorithms.

Supplementary Table S2 Reporting odds ratios and Information Components for the positive signals identified in FAERS associated with lumacaftor/Ivacaftor, using Kalydeco (ivacaftor) and Symdeko (tezacaftor/ivacaftor) as comparators.

Supplementary Table S3 Complete results of the sensitivity analysis excluding reports with concomitant medications reports.
